# A Rare Intersection: Managing Thrombotic Thrombocytopenic Purpura in the Context of Dengue Fever

**DOI:** 10.7759/cureus.68818

**Published:** 2024-09-06

**Authors:** Hamza Haj Mohamad, Abduljaleel M Toubah, Fatima Audi, Abdelrahman Nouh, Abdallah Jaber, Obaid Hashimi, Mahasin Shaheen

**Affiliations:** 1 Internal Medicine, Al Qassimi Hospital, Sharjah, ARE

**Keywords:** adamts-13, caplacizumab, dengue hemorrhagic fever, thrombotic microangiopathy, thrombotic thrombocytopenic purpura

## Abstract

Thrombotic thrombocytopenic purpura (TTP) is a rare, life-threatening condition that can lead to severe morbidity and mortality if untreated. This case report discusses a 31-year-old male with dengue fever who developed TTP, resulting in fatality despite timely diagnosis and comprehensive treatment. The patient presented with worsening symptoms, including body aches, gastrointestinal bleeding, and neurological issues. Initial treatment focused on managing dengue hemorrhagic fever, but TTP was later suspected, leading to the cessation of platelet transfusions and initiation of plasma exchange, steroids, and rituximab. Despite these efforts, the patient's condition deteriorated. This case underscores the challenges in managing TTP, especially when triggered by infections like dengue. The use of the PLASMIC score can be highly effective in suspecting TTP in these patients, allowing for the initiation of early management. While standard treatments include plasma exchange and immunosuppressive therapy, emerging treatments such as caplacizumab and the potential use of splenectomy may offer hope for better outcomes in the future.

## Introduction

Thrombotic thrombocytopenic purpura (TTP) is a rare and life-threatening condition characterized by the formation of microthrombi within small blood vessels, leading to hemolytic anemia, thrombocytopenia, and organ dysfunction. The incidence of TTP is approximately four to five cases per million people annually, affecting individuals across all age groups [1]. If left untreated, TTP can result in significant morbidity and mortality. The pathogenesis of TTP is primarily linked to a severe deficiency of the enzyme ADAMTS13, which is responsible for cleaving von Willebrand factor (vWF) multimers. This deficiency can be congenital or acquired, with the latter often resulting from autoimmune processes where autoantibodies inhibit ADAMTS13 activity [2].

Secondary TTP can arise due to various predisposing factors, including infections, malignancies, pregnancy, and certain medications. Dengue virus infection, known for causing dengue hemorrhagic fever, has rarely been associated with the development of secondary TTP [2]. In this case report, we present a rare and fatal instance of TTP secondary to dengue virus infection. Despite prompt diagnosis and aggressive management, the patient succumbed to the condition. This case highlights the diagnostic and therapeutic challenges posed by secondary TTP, particularly when triggered by infectious agents like dengue virus. Written informed consent was obtained from the patient’s next of kin for the publication of this case report.

## Case presentation

A 31-year-old male presented with fever, body weakness, and body pain. The patient, an ex-smoker and non-alcohol consumer, reported an eight-day history of fever initially diagnosed as dengue in private clinic. He received treatment at the clinic but returned with generalized body aches and central chest pain. He also reported passing fresh blood in his stools five days ago and experiencing intermittent mouth deviation and unilateral body weakness over the past five days, which resolved spontaneously.

The patient's vitals on admission were as follows: temperature of 36.5°C, heart rate of 90/min, respiratory rate of 17/min, blood pressure of 142/77 mmHg, and oxygen saturation (SpO2) of 98%. His body mass index (BMI) was 29.05. He appeared conscious and oriented but looked pale. Chest examination revealed clear lungs, cardiovascular examination indicated normal heart sounds (S1 and S2), abdominal examination showed right hypochondrial tenderness, and neurological examination revealed no deficits with power 5/5 in all limbs.

The patient's laboratory results indicated significant abnormalities as shown in Table 1. The patient had significant anemia with a low hemoglobin level and hematocrit. The platelet count was critically low at 6.0 x10³/µL. Markers such as total bilirubin, troponin-I, C-reactive protein (CRP), and lactate dehydrogenase (LDH) were markedly elevated, indicating liver dysfunction, myocardial injury, and systemic inflammation. Additionally, the patient tested positive for dengue virus by polymerase chain reaction (PCR) and immunoglobulin M (IgM) serology, confirming a concurrent dengue infection.

**Table 1:** Laboratory Results WBC: white blood cells; INR: international normalised ratio; ALT: alanine aminotransferase; CRP: C-reactive protein; LDH: lactate dehydrogenase; PCR: polymerase chain reaction; IgM: immunoglobulin M

Parameter	Result	Reference Range
WBC count (x10³/µL)	10.3	4.0 - 11.0
Hemoglobin (g/dL)	6	13.5 - 17.5
Hematocrit (%)	18.6	40 - 50
Platelet count (x10³/µL)	6	150 - 450
INR	0.9	0.8 - 1.2
Sodium (mmol/L)	137	135 - 145
Potassium (mmol/L)	2.7	3.5 - 5.0
Urea (mmol/L)	5.02	2.5 - 7.1
Total Bilirubin (µmol/L)	60.1	1.2 - 17.1
ALT (IU/L)	41	Jul-56
Troponin-I (ng/L)	1984	0 - 14
CRP (mg/L)	24.9	0 - 10
LDH (IU/L)	1646	85 - 227
Creatinine (µmol/L)	111	80 - 115
Dengue PCR	Positive	Negative
Dengue IgM	Positive	Negative

The ECG showed no abnormalities. The Chest X-ray was normal, revealing clear lung fields, clear costophrenic angles, and a normal cardiothoracic ratio. An ultrasound to the abdomen revealed normal findings.

The patient was diagnosed with dengue hemorrhagic fever (DHF). He was admitted for treatment that included antoprazole 40 mg IV every 12 hours, transfusion of six units of platelets, two units of fresh frozen plasma (FFP), and three units of blood, one unit at the admission and two units to be given if needed accordingly. The patient was also given two liters of IV fluids with 60 mmol KCL.

**Figure 1:**
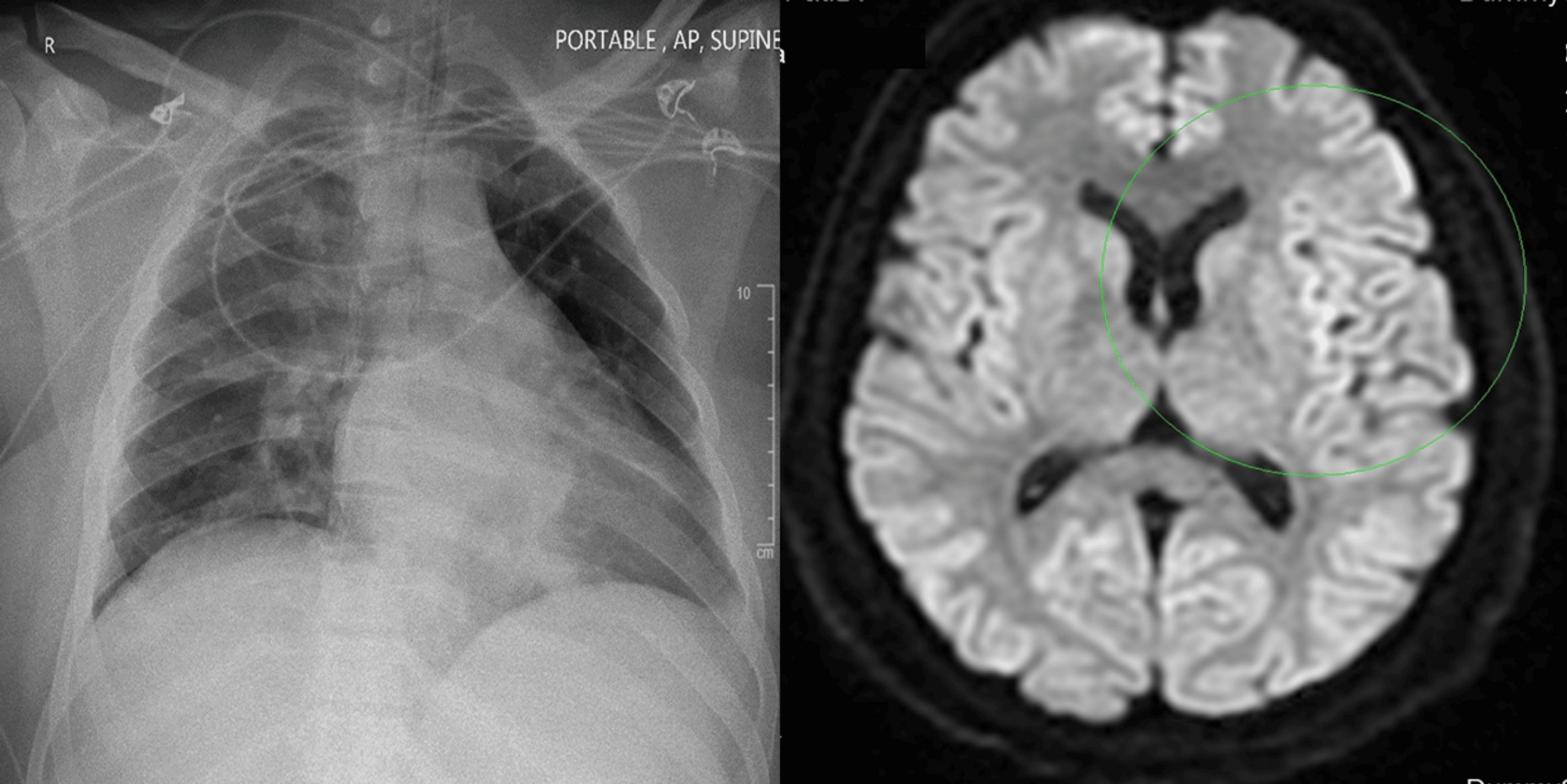
Imaging studies of the patient The picture on the left shows Chest X-ray demonstrating blunted left costophrenic angle. The picture on the right is MRI showing left fronto-parietal cortical subtle hyperintense diffusion-weighted imaging (DWI)–fluid-attenuated inversion recovery (FLAIR) signal (green circle)

On further clinical assessment, the patient was lethargic and had a generalized tonic-clonic seizure. Imaging studies, including a CT brain scan and MRI, indicated a mild hypoxic ischemic insult. While the magnetic resonance angiography (MRA) scan was clear, a repeated Chest X-ray showed a newly developed blunted left costophrenic angle. Both images are demonstrated in Figure 1. 

Repeated laboratory tests revealed features of microangiopathic hemolytic anemia with high indirect bilirubin and identifying schistocytes on blood smear. There was also severe thrombocytopenia with a platelet count 5.0 x10(3)/mcL even after platelet transfusion. More tests revealed high fibrinogen level, high D-dimer, elevated LDH, and increased reticulocyte count. The coagulation profile, including prothrombin time (PT), international normalised ratio (INR) and partial thromboplastin time (PTT), was normal and direct comb test was negative. Initially, the renal profile was normal but began to show deterioration with increasing creatinine.

Further hematology and nephrology consultation a day after the admission confirmed the presence of thrombotic microangiopathy by having microangiopathic hemolytic anemia with severe thrombocytopenia and identifying schistocytes on blood smear. A nephrology assessment was deemed necessary to exclude hemolytic uremic syndrome. ADAMTS13 activity and inhibitors had been requested three days after the admission, revealing low activity (<3%) and high inhibitors (112).

The ADAMTS13 results confirmed the diagnosis of acquired TTP and the patient was started on pulse steroids (IV 1g) and plasma exchange therapy (40ml\kg), which was planned to continue until improvement in the platelet count to more than 150,000 for two consecutive occasions, along with improvement in the LDH and renal function. Rituximab 375 mg/m2 every four days for four doses was initiated. A QuantiFERON Gold tuberculosis test was requested before starting rituximab.

Although in the case of severe ADAMTS13 deficiency, caplacizumab was recommended by our hematology team according to recent reports [2], but it was not available at the facility. A full autoimmune profile, including antinuclear antibody, double-stranded DNA, C3-C4, erythrocyte sedimentation rate, rheumatoid factor, anti-cyclic citrullinated peptide (CCP), antineutrophilic cytoplasmic antibody (ANCA), extractable nuclear antigens (ENA), and antiphospholipid workup, was requested to exclude autoimmune disorders.

The patient underwent approximately six sessions of plasma exchange and received pulse steroids for three days before being shifted to oral steroids. He also received the first dose of rituximab (825 mg) as per discussion with the clinical pharmacist. The second dose of rituximab was administered four days later with no significant improvement. Table 2 shows the therapeutic plasma exchange and laboratory data after each session. Despite these treatments, the patient continued to show evidence of hemolysis with high LDH levels and a high total bilirubin.

**Table 2:** Therapeutic plasma exchange (TPE) and laboratory data Hb: hemoglobin, HCT: hematocrit, LDH: lactate dehydrogenase

TPE	Hb (g/dL)	Platelet (x10³/µL)	HCT (%)	LDH (IU/L)	Total Bilirubin (µmol/L)	Creatinine (µmol/L)
1	8.4	13	24.8	1100	89.5	218
2	7.0	12	20.2	1054	64.5	183
3	7.9	27	21.8	909	69.9	155
4	8.6	27	24.7	936	51.6	158
5	8.9	44	24.8	1208	59.6	173
6	9.1	40	25.6	962	54.6	205

Meanwhile, a rapid response team (RRT) was announced as the patient became tachypneic, tachycardic, and in respiratory distress. The heart rate was between 140-145 beats per minute, and the respiratory rate was 26 breaths per minute. Chest examination revealed bilateral crackles up to the mid zones, although the Chest X-ray was unremarkable. The patient was placed on high-flow nasal cannula (HFNC) oxygen therapy and given a stat dose of furosemide 80 mg and metoprolol 2.5 mg for tachycardia. The patient was on two inotropic supports. After a nephrology review due to deterioration of creatinine levels as shown in Table 1, continuous renal replacement therapy (CRRT) was initiated. After that, he required intubation due to desaturation and hemodynamic instability.

During the seventh plasma exchange, the patient's oxygen saturation dropped to 70% and then to 66%. An urgent Chest X-ray revealed a total collapse of the left lung and right lobar collapse. Physiotherapy with suctioning and positioning the patient on the left side was attempted, but the patient continued to desaturate. A bedside bronchoscopy was performed, followed by chest toilet and suctioning. Positive end-expiratory pressure (PEEP) was increased to 10, resulting in an improvement in oxygen saturation to 97%.

Hours after that, the patient experienced a cardiac arrest in the form of pulseless electrical activity, leading to a Code Blue being announced. Cardiopulmonary resuscitation was initiated according to advanced cardiac life support protocol. Unfortunately, return of spontaneous circulation was not achieved, and the patient was declared dead due to thrombotic microangiopathy.

## Discussion

Thrombotic microangiopathy (TMA) leads to widespread blood vessel damage, causing hemolytic anemia, thrombocytopenia, and multi-organ failure, primarily impacting the kidneys, brain, and heart. Various forms of TMA include TTP, which is associated with a deficiency in ADAMTS13, an enzyme crucial for preventing abnormal clot formation. When ADAMTS13 is deficient, vWF accumulates, leading to excessive clotting and platelet consumption, resulting in organ dysfunction and bleeding risk [3]. TTP can be congenital, due to genetic mutations, or acquired, often triggered by autoantibodies from factors like medications, HIV, and pregnancy [4]. TTP is rare, with incidences between one and 13 cases per million, more common in women and typically presenting after age 40. Without treatment, mortality is 90%, but it drops to 10-15% with appropriate care [5,6].

Clinical manifestations of TTP are diverse. The classical pentad of hemolytic anemia, thrombocytopenia, fever, acute kidney injury, and severe neurologic symptoms is present in fewer than 5% of cases [7]. The PLASMIC score helps assess TTP likelihood, factoring in platelet count, hemolysis signs, mean corpuscular volume (MCV), INR, creatinine levels, and cancer or transplant status. Scores above 5 indicate high probability, with a 99% sensitivity [8]. Our patient's PLASMIC score was 7, indicating high likelihood of TTP. Confirmatory diagnosis involves ADAMTS13 activity assays and autoantibody testing. An activity level below 10% supports a TTP diagnosis but can also appear in severe sepsis and cancer, while the presence of ADAMTS13 inhibitors confirms that the cause of TTP is acquired [9]. Early diagnosis of TMA is crucial, as prompt identification and initiation of treatment significantly improve patient outcomes. Delays in recognizing TMA, especially in the context of overlapping conditions like dengue, can lead to rapid disease progression and increased mortality.

According to the 2020 Treatment Guidelines by the International Society on Thrombosis and Hemostasis [10], treatment of TTP involves plasma exchange as the cornerstone of therapy, typically involving the exchange of 40 to 60 mL/kg of plasma daily until clinical improvement is observed. Improvement is defined by normalized platelet counts, decreased LDH levels, and resolution of neurological symptoms. Plasma exchange should continue daily for at least two days after improvement is noted before tapering the frequency. If there is no improvement, exchanges may be performed twice daily.

Steroids significantly enhance treatment efficacy, with options including intravenous methylprednisolone (1g daily for three days) or oral prednisone (1-2 mg/kg daily) alongside plasma exchange. If there is inadequate response to these treatments, additional medications like rituximab (375 mg/m² weekly for two to eight doses) can be used. Rituximab may be initiated with plasma exchange or added if the disease is resistant to initial therapy [10]. Other drugs, such as vincristine, cyclosporine, azathioprine, and doxorubicin, are sometimes used but have limited supporting evidence regarding their effectiveness [10].

In our patient, two concurrent conditions, DHF and TTP, presented significant challenges. Initially, the patient received platelet transfusions on the first day of admission, which is standard practice for DHF. However, once TTP was suspected, the transfusions were promptly discontinued, as they are known to exacerbate TTP and increase mortality [11,12]. Despite undergoing seven plasma exchange sessions along with steroid therapy and rituximab, the patient's condition did not improve significantly, with persistently elevated LDH levels and minimal increases in platelet count. Although recent literature suggests that adding caplacizumab could be beneficial in treating TTP [2], this medication was unavailable at our institution. The unfortunate death of the patient has highlighted the need to explore new treatment options or adjunctive therapies for TTP, especially for cases resistant to standard plasma exchange and immunosuppressive treatments. One such consideration is splenectomy, which theoretically could reduce antibody production against ADAMTS13 by removing a primary site of antibody formation [13]. However, the effectiveness of splenectomy remains variable and requires further investigation.

A review of the literature reveals 17 cases of TMA triggered by dengue virus. Of these, nine were diagnosed as TTP, while the others were either hemolytic uremic syndrome (HUS) or unspecified TMA. Among the TTP cases, only one patient did not undergo plasma exchange, and this patient was the only one who died. The remaining cases showed varied outcomes, with most patients recovering completely. Treatment regimens included plasma exchange alone, plasma exchange combined with steroids in four cases, and the addition of rituximab in two cases. None of the reported cases did splenectomy or used other immunosuppressant medication. Table 3 summarizes and compares 17 reported cases of TMA triggered by dengue virus from the literature [14-29].

**Table 3:** summarizes and compares 17 reported cases of thrombotic microangiopathy (TMA) triggered by dengue virus from the literature * The diagnosis was based on clinical presentation, laboratory investigations, the presence of fragmented red blood cells in the blood film, elevated lactate dehydrogenase (LDH), and a rapid drop in hemoglobin. Biopsy was not performed nor was the ADAMTS13 test. HUS: hemolytic uremic syndrome; TTP: thrombotic thrombocytopenic purpura

Author	Year	Sex	Age in years	Main diagnostic test	Type of thrombotic microangiopathic	Treatment				Outcome
						Plasma exchange sessions	Steroids used	Rituximab	Other therapy	
Wiersinga et al. [14]	2006	Male	48	Renal Biopsy with normal von Willebrand factor-cleaving protease level	Atypical HUS	Received but number of sessions is not specified	No	No	Hemodialysis & antihypertensive drugs	Lost track
Rossi et al. [15]	2010	Male	45	Reduced level of ADAMTS13 activity (below 5% of normal) and positive autoantibodies against ADAMTS13.	TTP	11 cycles	No	No	No	Complete recovery
Hadianto and Mellyana [16]	2011	Male	8	Presumptive *	HUS	No	No	No	antihypertensive drugs	Complete recovery
Aroor et al. [17]	2014	Female	16	Presumptive *	HUS	8 cycles	No	No	fluid management and anti-hyperkalemic measures	Complete recovery
Deepanjali et al. [18]	2015	Female	25	Presumptive *	TTP	6 cycles	No	No	No	Complete recovery,
Bartholameuz et al. [19]	2016	Male	27	Presumptive *	TTP	1 cycle	No	No	No	Complete recovery
Ruslinda et al. [20]	2016	Male	25	Renal Biopsy	HUS	4 cycles	No	No	No	The patient ended up being dialysis-dependent with end-stage renal failure
Bhargava et al. [21]	2017	Male	32	Renal Biopsy	Thrombotic Microangiopathy	No	No	No	No	Complete recovery
Gavali et al. [22]	2017	Female	35	Presumptive *	TTP	8 cycles	Yes	Yes	No	Complete recovery
Nieto-Rıos et al. [23]	2017	Male	21	Normal ADAMTS13	Unspecified	13 cycle	yes	No	No	Complete recovery
Epelboin et al. [24]	2017	Female	43	Undetectable ADAMTS13 activity (<5% of normal) and positive anti-ADAMTS13 IgG autoantibodies.	TTP	No	Yes	No	Fresh frozen plasma	Death
Bastos et al. [25]	2018	Male	28	Very low ADAMTS13 activity (4%) and the presence of an inhibitor	TTP	6 cycles	Yes	No	No	Complete recovery
Gogireddy et al. [26]	2019	Male	2.5	Presumptive *	TTP	7 cycles	No	No	No	Complete recovery
Júnior et al. [27]	2021	Male	18	ADAMTS13 testing was performed, and the result was >100% and renal biopsy that showed mesangiolysis, endothelial edema, and fibrin deposition also the genetic testing revealed variants associated with atypical HUS.	Atypical HUS	No	No	No	Eculizumab	Required Renal Replacement Therapy (RRT) for a month after starting eculizumab but subsequently showed recovery of renal function
Júnior et al. [27]	2021	Male	28	Renal biopsy	Thrombotic Microangiopathy	No	No	No	antihypertensive drugs	Remission of hematuria and proteinuria and partial recovery of kidney function
Tee et al. [28]	2021	Male	29	Presumptive *	TTP	5 cycles	No	No	No	Complete recovery
Biswas et al. [29]	2023	Female	21	Presumptive *	TTP	8 cycles	Yes	Yes	No	Complete recovery

## Conclusions

In conclusion, TTP remains a challenging condition with current treatment modalities primarily focusing on plasma exchange, steroids, and rituximab. Despite these approaches, some patients experience inadequate responses, highlighting the urgent need for novel treatment strategies. Emerging options such as caplacizumab offer promise but are not universally available. Additionally, the role of splenectomy in TTP treatment has gained attention, with some literature suggesting its potential benefit by reducing antibody production against ADAMTS13. It is essential to maintain a high level of clinical suspicion for TMA, as this can lead to the early initiation of treatment, including empirical therapy, which may improve patient prognosis. Further research is required to validate the efficacy of these emerging treatments and to better understand the impact of splenectomy on TTP outcomes. Additionally, studies should explore whether the PLASMIC score can be relied upon for initiating early treatment without waiting for ADAMTS13 results.
